
JOURNAL INFORMATION
==============================
NLM Title Abbreviation: Crit Care
Journal ID: Crit Care
NLM Journal ID: 9801902

Critical Care

ISSN: 1364-8535
EISSN: 1466-609X
Publisher: BMC

ARTICLE INFORMATION
==============================
PMCID: PMC3891308
DOI: 10.1186/cc12644
Article ID: cc12644
Article version: 1
Subjects: Poster Presentation

Abstract withdrawn

Print publication date: 2013
Electronic publication date: 2013 Jun 19
Volume: 17
Issue: Suppl 3
Supplement: Seventh International Symposium on Intensive Care and Emergency Medicine for Latin AmericaPublication of this supplement was funded by the conference.
First page: P28
Last page: P28
Copyright: Copyright © 2013 licensee BioMed Central Ltd.
Copyright year: 2013
Copyright holder: licensee BioMed Central Ltd.
License: This is an Open Access article distributed under the terms of the Creative Commons Attribution License (http://creativecommons.org/licenses/by/2.0), which permits unrestricted use, distribution, and reproduction in any medium, provided the original work is properly cited.
License URL: https://creativecommons.org/licenses/by/2.0/

Conference date: 19-22 June 2013
Conference name: Seventh International Symposium on Intensive Care and Emergency Medicine for Latin America
Conference location: Sao Paulo, Brazil

==============================
*Critical Care* 2013, Volume 17 Suppl 3
http://ccforum.com/supplements/17/S3

CRITICAL CARE

MEETING ABSTRACTS

### Seventh International Symposium on Intensive Care and Emergency Medicine for Latin America

São Paulo, Brazil. 19–22 June 2013

Published: 19 June 2013

### Basic Science

### P44

### Impact of acute brain dysfunction on the outcomes of mechanically ventilated cancer patients

JIF Salluh[1,2], ICT Almeida[1], M Soares[1,2], FA Bozza[2,3], CR Shinotsuka[1,2], R Bujokas[1], VC Souza-Dantas[1], EW Ely[4,5]
[1]*Instituto Nacional de Câncer, Praça Cruz Vermelha, Rio De Janeiro, RJ, Brazil;* [2]*D'Or Institute for Research and Education, Rio de Janeiro, RJ, Brazil;* [3]*Instituto de Pesquisa Evandro Chagas, IPEC, Fundação Oswaldo Cruz, Rio de Janeiro, RJ, Brazil;* [4]*Vanderbilt University School of Medicine, Nashville, TN, USA;* [5]*Veteran's Affairs Tennessee Valley Geriatric Research Education Clinical Center, Nashville, TN, USA*
*Critical Care* 2013, **17(Suppl 3):**P44 (doi: 10.1186/cc12660)

**Introduction** Delirium and coma are a frequent source of morbidity for ICU patients. Several factors are associated with the prognosis of mechanically ventilated (MV) cancer patients, but no studies have evaluated delirium and coma (acute brain dysfunction). The present study evaluated the frequency and impact of acute brain dysfunction on hospital mortality.
**Methods** The study was performed at the National Cancer Institute, Rio de Janeiro, Brazil. We prospectively enrolled patients ventilated >48 hours with a diagnosis of cancer. The presence of acute brain dysfunction was assessed daily during the first 14 days of ICU care using the CAM-ICU. Patients were followed until hospital discharge. Univariate and multivariate analysis were performed to evaluate factors associated with hospital mortality.
**Results** A total 170 patients were included. Seventy-three percent had solid tumors, age 65 (53 to 72 (median, IQR 25 to 75%)) years. Admission SAPS II score was 54 (46 to 63) points and SOFA score was (7 (6 to 9)) points. Median duration of MV was 13 (6 to 21) days and ICU stay was 14 (7.5 to 22) days. ICU mortality was 54% and hospital mortality was 66%. Acute brain dysfunction was diagnosed in 161 patients (95%). Nonsurvivors had a higher frequency of acute brain dysfunction (110 (97.3%) vs. 51 (89.4%), $P = 0.06$). Survivors had more delirium/coma-free days (4 (1.5 to 6) vs. 1 (0 to 2), $P = 0.771$ (0.681 to 0.873), $P < 0.001$).
**Conclusion** Acute brain dysfunction in MV cancer patients is frequent and independently predictive of increased hospital mortality. Future studies should investigate means of preventing or mitigating acute brain dysfunction as they may have a significant impact on clinical outcomes.
**Acknowledgements** Supported by the National Cancer Institute, CNPq and FAPERJ.

### Sepsis

### P45

1. Sajid MS, *et al.*: *Sao Paulo Med J* 2009, **127:**231-237.
2. Buggy DJ, Crossley AWA: *Br J Anaesth* 2000, **84:**615-628.
3. Sessler DI: *Anesthesiology* 2001, **95:**531-543.

**Cite abstracts in this supplement using the relevant abstract number, e.g.:**
Santana AR, *et al.*: Role of hypothermia in the immediate postoperative period on mortality in a surgical ICU [abstract]. *Critical Care* 2013, **17(Suppl 3):**P59.
